# Novel Epigenetic Clock Biomarkers of Age-Related Macular Degeneration

**DOI:** 10.3389/fmed.2022.856853

**Published:** 2022-06-16

**Authors:** Saurav Mallik, Fran Grodstein, David A. Bennett, Demetrios G. Vavvas, Bernardo Lemos

**Affiliations:** ^1^Program in Molecular and Integrative Physiological Sciences, Department of Environmental Health, Harvard T. H. Chan School of Public Health, Boston, MA, United States; ^2^Rush Alzheimer’s Disease Center, Rush University Medical Center, Chicago, IL, United States; ^3^Ines and Frederick Yeatts Retina Research Laboratory, Retina Service, Department of Ophthalmology, Mass Eye and Ear, Harvard Medical School, Boston, MA, United States; ^4^Broad Institute of Harvard and MIT, Cambridge, MA, United States

**Keywords:** age related macular degeneration (AMD), age acceleration, age clocks, biomarkers, *GDF11*, retina, clocks, *FBN2*

## Abstract

Age-Related Macular Degeneration (AMD) is a bilateral ocular condition resulting in irreversible vision impairment caused by the progressive loss of photoreceptors in the macula, a region at the center of the retina. The progressive loss of photoreceptor is a key feature of dry AMD but not always wet AMD, though both forms of AMD can lead to loss of vision. Regression-based biological age clocks are one of the most promising biomarkers of aging but have not yet been used in AMD. Here we conducted analyses to identify regression-based biological age clocks for the retina and explored their use in AMD using transcriptomic data consisting of a total of 453 retina samples including 105 Minnesota Grading System (MGS) level 1 samples, 175 MGS level 2, 112 MGS level 3 and 61 MGS level 4 samples, as well as 167 fibroblast samples. The clocks yielded good separation among AMD samples with increasing severity score viz., MGS1-4, regardless of whether clocks were trained in retina tissue, dermal fibroblasts, or in combined datasets. Clock application to cultured fibroblasts, embryonic stem cells, and induced Pluripotent Stem Cells (iPSCs) were consistent with age reprograming in iPSCs. Moreover, clock application to *in vitro* neuronal differentiation suggests broader applications. Interesting, many of the age clock genes identified include known targets mechanistically linked to AMD and aging, such as GDF11, C16ORF72, and FBN2. This study provides new observations for retina age clocks and suggests new applications for monitoring *in vitro* neuronal differentiation. These clocks could provide useful markers for AMD monitoring and possible intervention, as well as potential targets for *in vitro* screens.

## Introduction

Age-related macular degeneration (AMD) is one of the leading causes of central vision loss among individuals aged 50 years and older (1–9). As the aging population increases, the increased prevalence of AMD continues to be a health concern. Globally, AMD was estimated to affect 196 million people in 2020 and is predicted to reach 288 million people by 2040 (3). AMD is classically divided in two categories. The non-exudative non-neovascular AMD (also known as “dry” AMD) affecting about 85% of the AMD population, and the exudative/neovascular AMD, also known as “wet” AMD affecting 15% of the AMD population. Though there has been good success in treating neovascular AMD (4, 10–12) the same cannot be told for the non-exudative “dry” AMD (6). Non-exudative AMD in its early and intermediate stages is characterized by sub-retinal pigment epithelium (RPE) deposits called drusen under the neuroretina (13) and RPE pigmentary variation with mild to moderate visual disturbances, while in its advanced atrophic stage is characterized by loss of photoreceptor/RPE/choriocapillaries complex and significant vision loss (9, 14, 15).

It is well accepted that AMD is complex multi-factorial disease (6, 9, 16–19). Despite successes in the identification of genes and molecular/cellular processes that contribute to AMD risk, mechanisms by which specific genetic variants contribute to AMD progression remain a matter of debate (20–27). Genetic studies have found over 35 genetic variants that increase risk of developing AMD, with many of those variants mapped to the complement system (28). In addition, variants pointed to lipid biology, turnover of the extracellular matrix (ECM) components, as well as inflammation as likely contributors to AMD pathogenesis. Other genetic variations connected to AMD include fibroblast growth factor 2, DNA excision repair protein, apolipoprotein E, and age-related maculopathy susceptibility protein 2.

Epigenetic biomarkers of aging include epigenetic clocks that are useful to estimate the biological age and that are built through measurements of DNA methylation, microRNAs, mRNAs as well as other epigenomic features. Epigenetic clocks are relevant for determining environmental and genetic factors that impact the aging process, for providing potential markers for disease monitoring, and to accelerate analyses of potential rejuvenating treatments. Research to identify biomarkers and predict biological age used data that ranged from DNA methylation to transcriptomics, microbiome, proteomics, frailty assessment, and neuro-imaging (5, 29, 30). Alterations in DNA methylation and RNA abundance have downstream impacts and play key regulatory roles; changes in RNA abundance are particularly pronounced in aging (31). These age-associated changes provide the raw material for novel aging biomarkers. For instance, DNA methylation age (DNAm age) were initially proposed from a set of age predictive CpG sites identified by elastic net penalized regression (32, 33). A positive or negative epigenetic age acceleration indicates an individual who is biologically older or younger than their chronological age. Assessment of biological age enabled the identification of individuals with substantial deviations from their chronological age and led to discoveries that accelerated biological aging in relation to diabetes, dementia, unhealthy behaviors, frailty, cancer, and more (34). Positive epigenetic age acceleration identifies individuals who are epigenetically older than their chronological age, while negative epigenetic age acceleration identifies individuals who are epigenetically younger than their chronological age.

A variety of models are able to ascertain association between expression/methylation patterns and age (32, 33, 35–39) but epigenetic clock applications to AMD and to *in vitro* neuronal differentiation has not been fully addressed. Hunter et al. found hypermethylation of Glutathione S-transferase isoform mu1 (GSTM1) and mu5 (GSTM5) promoter in RPE cells from AMD donor eyes when compared to control (40). Wei et al. (41) and Oliver et al. (42) found conflicting information about the methylation status of IL17RC in peripheral blood mononuclear cells from AMD and control patients (41, 42). A single genome-wide epigenetic study of AMD reported hypomethylation at the ARMS2/HTRA1 locus and hypermethylation at the protease serine 50 (PRSS50) locus compared to controls (43). Wang et al. (44) using ATAC-Seq analysis revealed a widespread decrease of chromatin accessibility in RPE cells from AMD patients (44). Vallée et al. (45) provided a survey that highlighted the importance of circadian rhythm dysregulation in exudative (wet) AMD by abnormal upregulation of the canonical WNT/β-catenin pathway (45). Ratnapriya et al. (46) conducted a study on the genetic landscape of AMD and built an Eye Genotype Expression (EyeGEx) database for the post-GWAS-based interpretation of ocular traits (46). Brooks et al. (47) performed an interesting analysis to determine the regulatory signals deficient in building retinal organoids and yielded the experimental validation through generating a mature retina *in vitro*, hence facilitating research in the disease modeling and evaluation of therapeutic interventions (47).

Here we developed age clocks for the retina and explored their utilization in AMD cases with increasing severity. The proposed clocks yielded good separation among AMD samples with increasing severity score (Minnesota Grading System scores 1–4 or, MGS scores 1–4), regardless of whether clocks had been trained in retina samples, dermal fibroblast samples, or in combined retina-dermal datasets. In addition, clock application to other related cells (viz., embryonic stem cells and iPSC cells) confirmed age reprograming in IPSCs. Interestingly, model application to *in vitro* neuronal differentiation points to broader applications. All in all, our study provides new observations of retina age clocks and their underlying genes that might be helpful to understand the possible causes and factors for retinal aging and age-associated eye diseases.

## Materials and Methods

### Data Source

We first utilized human retina gene expression human data (NCBI Gene Omnibus ID: GSE115828) collected with Illumina HiSeq 2500 (38, 46, 47). The whole gene expression data matrix consisted of a total of 18,053 unique genes and a total of 453 MGS samples. There were four categories/levels of MGS samples: 105 MGS level 1 (control) samples, 175 MGS level 2 (diseased), 112 MGS level 3 (diseased) and 61 MGS level 4 (diseased) samples. The Minnesota Grading System (MGS) score identifies the severity of AMD (48, 49). MGS1 donor retinas demonstrated no AMD features and serve as control, whereas MGS2 to MGS4 samples represent progressively more severe disease stages. In addition, we utilized three more datasets, viz., Dermal fibroblast dataset, primary fibroblast dataset and neuronal differentiation dataset. Dermal fibroblast data (NCBI Gene Omnibus ID: GSE113957) consisted of 27,142 genes and 143 samples. Primary fibroblast data (Gene Omnibus ID: GSE97265) contained 6,732 genes and 14 samples, while the neuronal differentiation data (Gene Omnibus ID: GSE56796) consisted of 44,562 initial genes and 24 samples.

### Detecting Outlier Genes Through Density Based Clustering of Applications With Reducing Noise

We applied a well-known noise removal cluster algorithm, “Density Based Clustering of Applications with Reducing Noise” (DBSCAN) (50, 51) using the Set of initial feature/gene vectors. Outlier features were omitted from further analyses. Specifically, we initially estimated the knee point using kNN distance plot; the determined knee value was utilized as the corresponding eps-neighborhood value. Other parameters used were default setting parameters. This produced some density-based clusters, while each cluster comprised of (i) core features, (ii) border features and (iii) noisy features. Thereafter, we removed those noisy features from the data. The noise-free features were then used for the next step (cross validation and regression analysis). The cluster plot generated by DBSCAN clustering technique was examined. In DBSCAN clustering algorithm, two necessary user-defined parameters were termed as (i) epsilon (*eps*) and (ii) minimum points (*MinPts*). The *eps* that was the radius of the neighborhood around any point, was termed as epsilon-neighborhood (e-neighborhood) of that point, while *MinPts* was the minimum number of neighbors inside eps. Whenever a point had a neighbor count score that was higher than or equal to *MinPts*, the point is stated as a core point. If the number of the neighbors of any point was less than *MinPts*, but the point belonged to the e-neighborhood of a core point, the point was stated as border point. When a point was neither a core point nor a border point, that point was treated as a noisy/outlier point. Our goal was here to determine the dense regions which could be estimated through the number of points/objects close to a specific point. Here initially we determined the knee-point by K-nearest neighbor (KNN) distance plot. KNN distances were calculated and sorted to estimate the knee-point. After that, these were scaled to range between 0 and 1, and the derivative was evaluated. The first point in which the derivative was higher than a certain value (say, 1), was treated as the knee-point. The scaled distance score of the knee-point was termed eps-neighborhood value.

For the case of retina data with full feature set, we applied kNN distance plot with the data while k was set as the sample size plus 1 (= 453 samples +1). Here we fixed height “*h”* as 5,000 in the plot to determine the knee point. That knee was used as eps score in the next step i.e., DBSCAN clustering to identify the outlier features. Using DBSCAN clustering, we obtained 75 noisy features (outliers) and omitted them from the further analysis. We then utilized the remaining noise-free features (= 17,978) for clock model development. For the full feature set of dermal fibroblast data, using DBSCAN clustering, we identified 58 noisy features and discarded them from the further analysis. We then utilized the remaining noise-free features (= 14,825) for clock model development.

### Building Age Clock Model Through Glmnet Regression

After outlier detection through DBSCAN, data sets were processed for further analysis. We utilized leave one out cross-validation (LOOCV) in specific datasets (e.g., retina tissue samples) to divide a dataset into training and test sets. In LOOCV, only one sample would be used as test set, while remaining samples belonged to the training set. That step would be repeated for each of the remaining samples. We chose the subset of the expression data containing only those training samples, and the age of those training samples from clinical data. On the other hand, we also selected the subset of the expression data consisting of only those test samples, and the age of those test samples from clinical data.

After identification of training and test samples through LOOCV, we applied “glmnet” regression R tool (52–54) on the training expression data as response variable and logarithm transformed training aging data as predicted variable, and then generated a lambda (λ) value whereas classification error was equal to the least square error (lse). Here the target function T for glmnet regression technique that utilized a mixed linear model, is generally a single-objective function. The respective model determined a set of coefficients (signifying a set of selected features/genes) for which the value of T would be minimized. T was defined in the following:


$$T=\frac{1}{2\,m}\,\sum_{i=1}^{m}{\left(y_{i}-\sum_{j=1}^{n}\beta_{i}\,{\text{x}}_{i\,j}-\beta_{0}\right)}^{2}$$

+λ(1-α2∑j=1nβ+j2α∑j=1n|βj|),

where m referred to as the number of samples and n denoted the number of features. Here x_*ij*_ was termed as the gene expression profile at i-th sample and j-th feature, whereas y_*i*_ was the logarithm converted chronological age of i-th sample. The combination of lasso and ridge regulation methods with each having equal preference had been utilized here for the inclusion an additional penalty (also called as extra constraint) to the coefficients of predicted variables. Here λ (> 0) was basically a tuning parameter that helps to regulate the whole penalty against the respective coefficients, whereas α was another tuning parameter whose ranges lied in between 0 and 1 (0 < α < 1) revealing the compromise between ridge technique (α = 0) and lasso methodology (α = 1). We fixed α to 0.5. λ was chosen through LOOCV (i.e., *m*-fold cross-validation, while *m* be the number of sample size) internally by following the one-standard-error policy. After determining the specific λ, we selected features and obtained their coefficients depending upon the estimated λ. Thereafter, we discarded those features whose coefficient was zero. And then we continued with remaining non-zero features for age prediction.

For the full feature set of retina data, we used MGS level 1 samples (control samples) to build age clock models. We applied LOOCV for splitting the data into training set and test set. In LOOCV, only one sample would be utilized as test set and remaining 104 samples belonged to the training set. That step was repeated for each of the remaining samples. For training, we selected the subset of the data consisting of only those training samples, and the age of those training samples from clinical data. Similarly, for the test model, we picked up the subset of the transcriptomic data having only those test samples, and the age of those test samples from the clinical data. After determination of training and test samples with LOOCV, we used “glmnet” regression on the training methylation data having 104 samples and 17,978 features as response variable and logarithm transformed training aging data having chronological ages of 104 samples as predicted variable, and then determined a lambda (λ) score for which classification error was equal to the least square error (lse). The corresponding regression model computed coefficients for all features among which non-zero coefficients (i.e., feature selection) will be identified. These corresponding features having non-zero coefficients were termed as selected features for each model. In addition, we computed the occurrence of each gene in all those evolved integrated models. Similarly, for the restricted feature set of retina data, we followed the same pipeline (LOOCV and “glmnet,” consecutively) while working on training data containing 104 MGS1 samples and 5,321 features, and test data consisting of only one MGS1 sample and 5,321 features during the iteration of “glmnet” regression.

For the restricted feature set of dermal fibroblast data, using the 143 samples, we utilized LOOCV first and then applied glmnet regression on the training expression data containing 142 samples and 5,321 features as response variable and logarithm transformed training aging data having chronological ages of 143 samples as predicted variable, and then determined a λ-score for which classification error was equal to the least square error. The regression model computed coefficients for all genes of which non-zero coefficients (i.e., gene selection) will be identified. Those genes having the non-zero coefficients were behaved as the selected features for each evolved model.

For the restricted feature set of joint data, using the 248 samples, we utilized LOOCV to split the entire data into training set and test set. In LOOCV, only one sample was treated as test set while remaining 247 samples was used as the training set. We repeated that step for each remaining sample (i.e., the repetitions of total 248 times). After LOOCV, we applied “glmnet” regression methodology on the training methylation data containing 247 samples and 5,321 features as response variable and logarithm transformed training aging data having chronological ages of 247 samples as predicted variable, and then determined a λ-score for which classification error was equal to the least square error (lse). The regression model computed coefficients for all genes of which non-zero coefficients (i.e., gene selection) will be identified. Genes having non-zero coefficients were selected as features in each model.

### Evaluating Age Clocks

**FIGURE 1:**
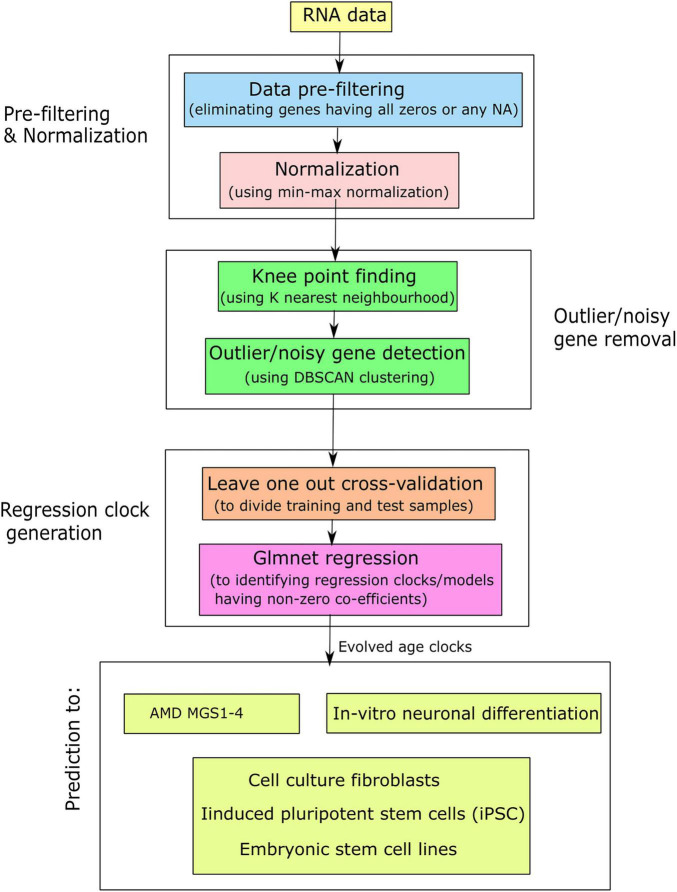
Simplified flowchart for the generation of aging clocks and their application in AMD and related contexts.

After generating the evolved age clocks from each kind of data, we applied on other data samples to predict them using the model coefficients. Here we mainly utilized four evaluation metrics: (i) age acceleration (AA), (ii) Median absolute error (MAE), (iii) Spearman’s correlation (55), and (iv) gene occurrence. Age acceleration (AA) is stated as the difference between predicted age and chronological (original) age. AA is the residual following regression of predicted (biological) age on chronological age. A positive age acceleration represents predicted age higher (older) than chronological age, while a negative age acceleration represents predicted age lower (younger) than chronological age. MAA is defined as the median score of age acceleration of a set of samples. MAE (56, 57) is defined as the median value of all absolute differences between the predicted and targeted scores. MAE was defined as follows: *MAE*(ŷ,*y*) = *med*(|ŷ1−*y*_1_|,…,|ŷ*m*−*y*_*m*_|), where *med* was median score, ŷ_*i*_ referred to the predicted score of the i-th sample and y_*i*_ denoted the respective true (original) score. MAE was a minimization objective (i.e., lower value of MAE is favored). Spearman’s correlation is well-known linear similarity finding method that is useful here to determine the similarity between predicted age vectors and original (chronological) age vectors, while gene occurrence (frequency) is termed as the number of times it appears among all regression clock generation. Here occurrence (in %) = occurrence*100/total number of clocks. In addition, rank wise cumulative sum of occurrence for each gene was computed. Here, rank-wise cumulative sum of occurrence (in %) = rank wise cumulative sum of occurrence * 100/total number of events, where “total number of events” denotes the summation of occurrence of all initially participating genes during clock generation. Finally, we compared the different regression models through the intersection of non-zero occurrence genes. We used Venn diagrams to represent this intersection. Figure 1 shows a simplified flowchart of our approach.

## Results

### Retina Age Clocks

We initiated our analysis with transcriptome data consisting of 18,053 features/genes and 453 Retina samples (see section “Materials and Methods”). The set includes control samples (MGS1) and three levels of AMD severity (i.e., MGS2-MGS4, with MGS4 representing the most severe AMD pathology). After quality control and noise removal (see section “Materials and Methods”), we carried forward 17,978 features, and applied leave one out cross-validation (LOOCV) to build 104 age clock models using MGS 1 samples (i.e., retina control samples). For each model, we computed the absolute error (AE) and age acceleration (AA) for the test sample of the LOOCV. For MGS 1 samples, we identified 49 individuals with positive age acceleration (AA^+^) and 56 individuals with negative age acceleration (AA^–^). This ∼50% positive/negative age acceleration fits the expectation for a training set. The maximum value of AA was +20.4 years while the minimum value of AA was –19.1 years. The model for which we obtained the maximum AA had 94 features/genes, while the model for which we identified the minimum AA had 54 features/genes. The model yielding the lowest absolute AE of 0.003 consisted of 78 features/genes.

We built age clock models using a restricted set of 5,321 features comprising genes that are (i) evolutionarily conserved between human and mouse, and (ii) shared across all datasets used in this study. Using this conserved and conservative feature set, we identified 51 samples with positive age acceleration (AA^+^), and 54 samples with negative age acceleration (AA^–^). The maximum value of AA was 18.5 while minimum value of AA was –17.4. The model for which we obtained the maximum AA used 64 features, while the model for which we identified the minimum AA used 98 features. The model yielding the lowest AE (0.07) used 80 features. We conclude that the smaller set of evolutionarily conserved genes yielded age clock models that were comparable to those built with the larger gene set.

**FIGURE 2:**
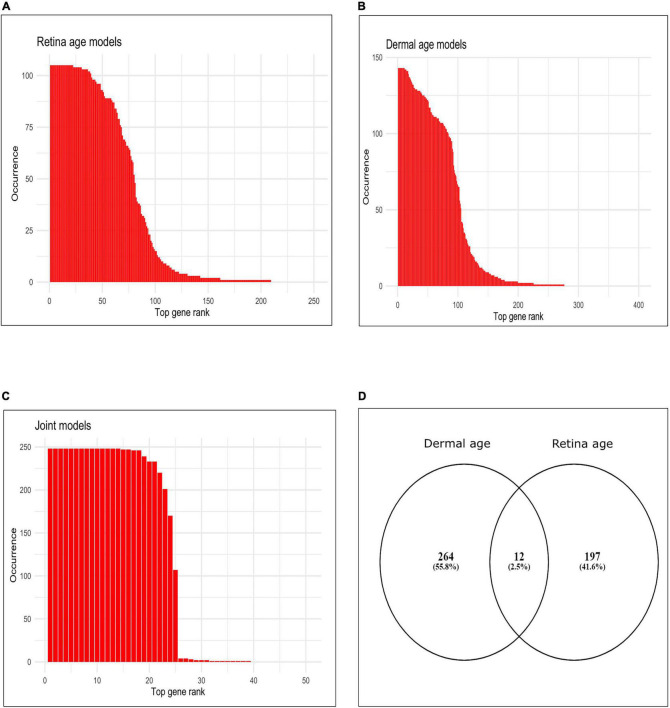
Occurrence of top genes in age clocks models. **(A)** Bar plot of occurrence of top 250 genes most represented in clocks using retina tissue (AMD MGS1, conserved feature set), **(B)** bar plot of occurrence of top 400 genes most represented in clocks using dermal fibroblast samples, **(C)** bar plot of occurrence of top 50 genes most represented in clocks developed using combined retina-dermal datasets, **(D)** Venn diagram of genes represented in retina and dermal fibroblast clocks.

We assessed gene occurrence in the 105 AMD MGS1 clocks both built with the conserved genes (Figure 2A). Out of all 5,321 input genes, 209 genes were represented in at least one age clock model (i.e., occurrence = 1). The total number of gene occurrence events across all 105 models was 8,354. The top 200 most frequent genes accounted for 99.89% of all events (i.e., rank wise cumulative sum of occurrence * 100/total number of events). The top 79 most frequent genes were represented in over 50% of all models (= occurrence * 100/total number of models) and accounted for 88.32% of all events. The top 22 genes which were represented in all models (100% occurrence) were *CFLAR, HSD17B6, DGKA, MYLK, FGF10, ARG2, PDPR, PALMD, PCDHB2, GORASP2, SNX19, BMP4, DTNA, RHOB, LYPD1, APBB2, GALNT10, CRADD, PARD6G, GJC1, THBS2, NEURL1B*.

### Application of Retina Age Clocks to Age-Related Macular Degeneration

**FIGURE 3:**
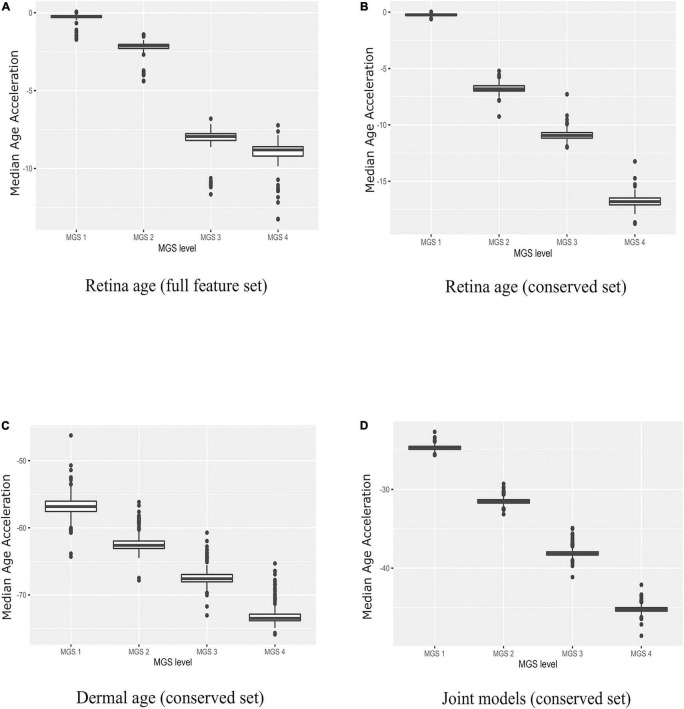
Boxplots of age acceleration in AMD MGS1-4 individuals. **(A)** Retina age clock models (AMD MGS1, full feature set), **(B)** retina age clock models (AMD MGS1, conserved feature set), **(C)** dermal fibroblast age clock models (conserved set), and **(D)** combined retina-dermal age clock models (conserved set). Shown are median age acceleration for each retina group (AMD MGS1-4) for the different clock models. All four groups are significantly different (*P <* 0.001).

Next, we applied all 210 retinal age clocks (105 clocks developed with the full gene set and 105 clocks developed with the conserved gene set) to the MGS1-4 samples, and computed model wise Median Absolute Error (MAE) and model wise Median of Age Acceleration (MAA). For MGS 1, negative median age acceleration (MAA^–^) was observed for most of the models (= 206 models), while positive median age acceleration (MAA^+^) was only observed for 4 models. Minimum value of model wise MAA was found as –1.72, while maximum value of model wise MAA was 0.06. Minimum value of model wise MAE was 0.91, while maximum value of model wise MAE was 7.61. For AMD MGS 2 samples, negative median age acceleration (MAA) was observed for all models. Minimum value of model wise MAA was found as –4.39 years, while maximum value of model wise MAA was –1.39 years. The minimum and maximum values of model wise MAE was 5.12 and 7.36 years, respectively. For AMD MGS 3 samples, MAA ranged from –11.66 to –6.80 years; MAE ranged from 7.30 to 12.12 years. For AMD MGS 4 samples, MAA ranged from –13.26 to –7.23 years; MAE ranged from 8.53 to 13.26 years (Supplementary Figure 1A). AMD MGS label-wise box plot of MAA using all 105 evolved retina (AMD MGS1-based) age models for the all-feature set and the conserve set are depicted in Figures 3A,B, respectively. See Supplementary Figures 1, 2 for data from each model. Analyses with models generated with the full gene set or with the smaller set of evolutionarily conserved genes yielded very similar results. We conclude that samples with more advanced AMD pathology score show increasingly large negative age acceleration (*P* < 0.0001 for all cases, ANOVA).

### Dermal Fibroblast Age Clocks

We explored the possibility that age clock models built with skin samples could be informative about AMD. To address the issue, we focused on the set of 5,321 evolutionarily conserved genes and 143 dermal fibroblast samples from donors of known age (see section “Materials and Methods”). In the testing phase we built 143 models with LOOCV. The analyses yielded 69 samples with positive median age acceleration (MAA+) and 74 samples with negative median of age acceleration (MAA–). We estimated MAE as 3.49, considering all 143 dermal fibroblast models together. For the prediction of all 143 dermal fibroblast samples themselves, the model-wise MAE values ranged (2.24, 9.36), thus the minimum MAE value (= 2.24) was extremely good. The model-wise Spearman’s rank correlation (rho) between the vector of predicted age and the vector of chronological age of dermal fibroblast samples for each model ranged from 0.89 to 0.99.

Finally, we computed the frequency (occurrence) of each gene (feature) in all 143 dermal fibroblast models (Figure 2B). The total number of events for 143 models was 13,25. Out of all 5,321 input genes and 143 models, 276 genes were represented in at least one model (i.e., frequency = 1). Top 200 most frequent genes were accounted for 99.24% of all events (i.e., rank wise cumulative sum of occurrence * 100/total number of events). Top 97 most frequent genes were selected over 50% occurrence in all models (= occurrence*100/total number of models) and accounted for 86.90% of all events. Interestingly, 11 genes were represented in all models (i.e., 100% occurrence). These were *MLLT11, CNKSR3, FAHD2B, FBN2, ALKBH3, FAS, SLC22A15, RPN2, BST2, XPNPEP1, CCDC8*.

### Application of Dermal Fibroblast Age Clocks to Age-Related Macular Degeneration

We used the dermal fibroblast clocks to predict age in the retina of MGS1-4 samples (Figure 3C). All 143 models yielded negative MAA in all retina samples, with absolute MAE that ranged from 46.23 years in MGS1 to 65.31 years in MGS4. Notwithstanding these larger MAEs, the skin-based age models replicated the same pattern we observed for retina-based models and readily separated retina samples in accordance with MGS pathological score (Figure 3C). The range of model-wise MAA of MGS1 samples for dermal fibroblast models was between –64.29 and –46.23. The range of model-wise MAA of all MGS4 samples for dermal fibroblast models was between –75.89 and –65.31 (MGS 1-4 box plot of MAE and model-wise line plots of MAA and MAE using all those Dermal fibroblast age clocks are shown in Supplementary Figure 3). Finally, we compared gene occurrence in the dermal fibroblast clocks and retina MGS1 clocks and identified 12 common (Figure 2D). Those genes were *RHOJ, VTI1A, CDCA7, LYPD1, PRSS12, MEST, LGR5, TMEM132B, PCDHB2, ARSK, FAT1, BMP4*.

### Combined Retina-Dermal Clocks and Application to Age-Related Macular Degeneration

Here we pursued integrated models using both retina and dermal fibroblast samples for training (i.e., 143 dermal fibroblast and 105 AMD MGS1 samples together). We normalized the evolutionarily conserved datasets gene wise using min-max normalization and produced a combined set of 248 samples that included both the 143 dermal fibroblast samples and 105 MGS1 samples. In the testing phase we built 248 models with LOOCV. We observed 88 samples with positive age acceleration (AA+) and 160 samples with negative age acceleration (AA-). The MAE value considering all 248 integrated models together was 22.02 years. In addition, we evaluated model performance in the retina and dermal samples separately. For the prediction of all 143 dermal fibroblast samples themselves, the model-wise predicted MAE values ranged (12.65, 15.86). Spearman’s rank correlation between chronological age of dermal fibroblast samples and predicted age were generally high (range: 0.77–0.81). Thus, for the dermal fibroblast samples, the integrated model fits reasonably well. For the prediction of all 105 MGS1 samples, the model-wise predicted MAE values range (22.68–25.64) was nearly twice as large as the MAE for skin samples. MAEs were larger for MGS4 samples (range: 42.12–48.59). Noteworthy, joint dermal-retina models replicated the same pattern we observed for retina-only and skin-only models, and readily separated retina samples in accordance with MGS pathological score (Figure 3D and Supplementary Figure 4).

In addition, we estimated the occurrence of each gene in all 248 integrated models. Here total number of events for 248 models was 5,886. Out of all 5,321 input genes, 39 genes were represented in at least one clock (i.e., frequency ≥ 1). Top 38 most frequent genes were accounted for 99.98% of all events (i.e., rank wise cumulative sum of occurrence * 100/total number of events). Top 24 most frequent genes were selected over 50% occurrence in all models (= occurrence * 100/total number of models) and accounted for 97.76% of all events. Interestingly, the top 14 genes that had 100% occurrence, were *SYNE2, LNX1, VDR, PLCG1, BCAS4, RPAIN, GDF11, FBN2, CPNE8, ALKBH3, MYO1D, ZNF518B, TEAD4, GPC2*. We provided the occurrence plot of the top 50 ranked genes in the rank wise manner in Figure 2C.

We found 25 genes shared between clocks developed with dermal fibroblast and clocks developed with combined retina-dermal fibroblast datasets, viz., *MLLT11, FBN2, ALKBH3, FAS, BST2, XPNPEP1, GPC2, ZNF518B, TEAD2, SYNE2, DUSP2, CPNE8, GDF11, C16orf72, CKAP4, TEAD4, PEG10, BCAS4, APBB1IP, SEMA4C, RIN2, LNX1, RNF149, EXOC8, RPAIN*, and 2 genes shared between retina MGS1 and joint retina-dermal clocks, viz., *LAMA4* and *MYO1D*. Interestingly, literature evidence has implicated some of these genes in AMD (viz., *C16orf72, GDF11, FBN2*) (58–61).

### Application of Age Clocks to Reprogrammed Primary Fibroblasts

**FIGURE 4:**
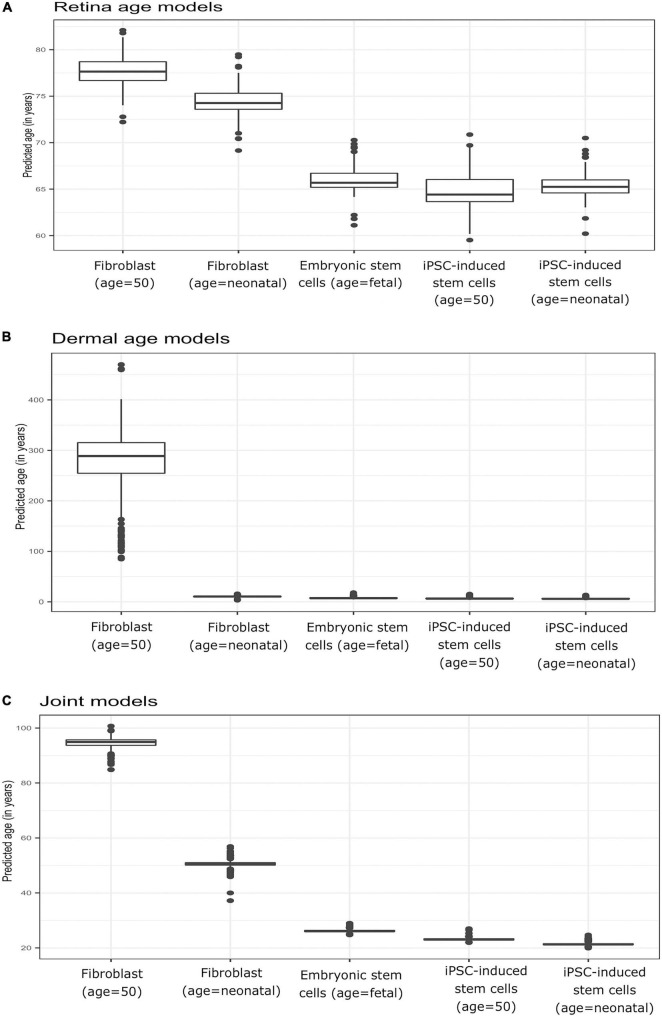
Boxplots of predicted age (group-wise) in Primary fibroblast samples, primary embryonic stem cells, and induced pluripotent stem cells (iPSC). Shown are estimates obtained with clocks developed using **(A)** retina (AMD MGS1) tissue, **(B)** dermal fibroblast samples, and **(C)** combined retina-dermal datasets for the set of 5,321 common genes. The five groups consisted of fibroblasts from neonatal individuals (FN1, FN2), induced pluripotent stem cells (iPSC) derived from fibroblasts from neonatal individuals (IN2.1, IN2.2, IN2.4, IN2.5), fibroblasts from 50 year old individuals (F50, F50S), induced pluripotent stem cells (iPSC) derived from fibroblasts from 50 year old individuals (I50.2, I50.3, I50S.1, I50S.2) and embryonic stem cells (H1, H9) samples, respectively.

To verify model behavior, we assessed the ability of our clock models to predict age in cell culture fibroblasts, reprogrammed primary fibroblasts, and embryonic stem cell lines. We used a dataset comprised of two neonatal samples (age = 0, FN1, FN2) and their respective iPSC (IN2.1, IN2.2, IN2.4, IN2), two samples from a 50-year-old individual (F50, F50S) and their respective iPSC (I50.2, I50.3, I50S.1, I50S.2), and two embryonic stem cell lines (H1 and H9). In these samples, we computed the average predicted age across all models of a given type (retina tissue clock, dermal clocks, or clocks with combined retina-dermal samples). Reassuringly, all models displayed similar overall behavior across samples (Figures 4A–C), with similar age estimates for embryonic stem cells and iPSCs derived from fibroblasts obtained from neonatal or 50-year-old individuals. However, dermal age models were more accurate with age estimates for embryonic stem cells and iPSCs that were closer to zero. Clocks developed with dermal samples also yielded age estimates for neonatal fibroblasts that were numerically closer to zero relative to estimates obtained with clocks developed with retina tissues. Similarly, all models over-estimated fibroblast age from the 50-year-old donor. Specifically, for clocks estimated with dermal tissues we obtained 10.49 years as the average predicted age for neonatal fibroblasts (FN1, FN2), 6.45 years for iPSCs derived from neonatal fibroblasts (IN2.1, IN2.2, IN2.4, IN2.5), 269.66 years for 50-year old fibroblasts (F50, F50S), 6.96 years for iPSCs derived from 50 year old fibroblasts (I50.2, I50.3, I50S.1, I50S.2), and 7.88 years for embryonic stem cells (H1, H9). For retina AMD MGS1 clocks, we obtained 74.41 years as the average predicted age for neonatal fibroblasts (FN1, FN2), 65.36 years for iPSCs derived from neonatal fibroblasts (IN2.1, IN2.2, IN2.4, IN2.5), 77.62 years for 50-year old fibroblasts (F50, F50S), 64.83 years for iPSCs derived from 50 year old fibroblasts (I50.2, I50.3, I50S.1, I50S.2) and 66 years for embryonic stem cells (H1, H9). For the 248 clocks trained on retina (MGS1) and dermal samples together, we observed 50.59 years as the average predicted age for neonatal fibroblasts (FN1, FN2), 21.43 years for iPSCs derived from neonatal fibroblasts (IN2.1, IN2.2, IN2.4, IN2.5), 94.501 years for 50-year old fibroblasts (F50, F50S), 23.20 years for iPSCs derived from 50 year old fibroblasts (I50.2, I50.3, I50S.1, I50S.2) and 26.22 years for embryonic stem cells [H1, H9]. Collectively, these observations indicate that age clock model yield reasonable age estimates that are consistent with reprogramming.

### Application of Retina Clocks to *in-vitro* Neuronal Differentiation

**FIGURE 5:**
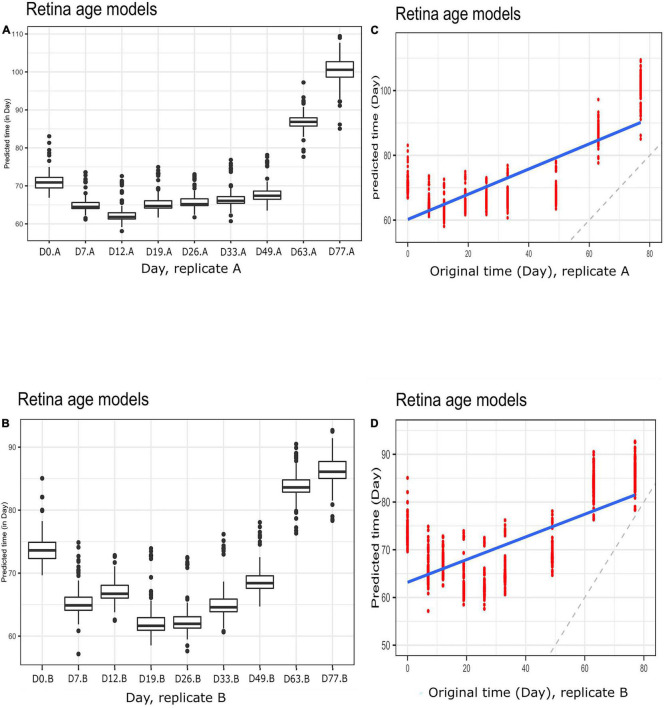
Boxplots of predicted time (clock age in days) during neuronal differentiation. **(A)** Group A replicates, **(B)** group B replicates. Regression plots showing predicted time (age) and original differentiation time for **(C)** group A replicates, **(D)** group B replicates. Shown are data for neuronal differentiation samples and retina (AMD MGS1) age models. Note the discrepancy between actual ages (in days) and clock age estimates (in days).

**FIGURE 6:**
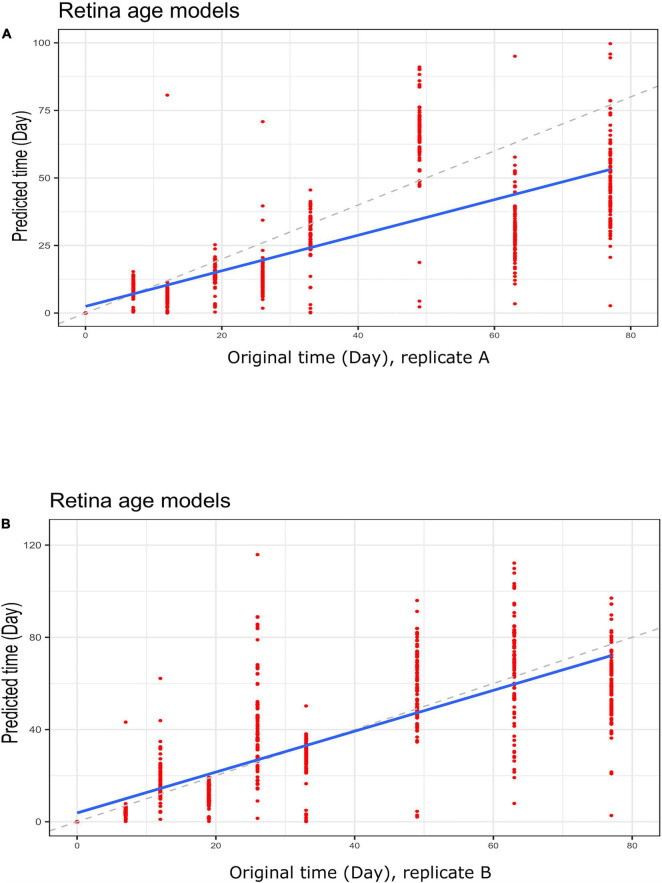
Regression plots showing predicted time (clock age in days) and original differentiation time during neuronal differentiation. Data for **(A)** group A replicates and **(B)** group B replicates. Shown are data for retina (AMD MGS1) age models using a LOOCV approach to re-adjust model coefficients. Note a much closer correspondence between differentiation age (in days) and clock age estimates (in days).

To gain further insight into our clocks we also observed model behavior through *in vitro* neuronal differentiation. To address the issue, we used a dataset consisting of a time course of cellular differentiation from neuronal stem cells to neurons. While we did not expect models trained on chronological age in years to be directly applicable to neuronal differentiation in days, a correlation between predicted age and differentiation time might be expected. Indeed, the retina models did not numerically recapitulate differentiation day; however, we noted a relative increase in “age” from day 19 to day 77 that was reproducible in two replicates (for biological replicates group A and B samples) (Figures 5A,B and Supplementary Figure 5; Figures 5C,D for corresponding regression plots). Furthermore, the correlations between differentiation day and predicted age were significant and surprisingly high; the models yielded the following correlations between the original differentiation day and the predicted “age”: 0.54 and 0.41 for A and B replicates, respectively, across the full differentiation span. Next, we conducted another experiment of readjustment of co-efficient (i.e., two times LOOCV) procedure to predict differentiation day. Here a second round of LOOCV was conducted using features (i.e., genes) previously selected for the retina clocks. As expected, the predicted time (in days) was a much better reflection of the original differentiation day after a second round of optimization (i.e., the predicted regression line was close to the optimal dotted line) (Figures 6A,B and Supplementary Figure 6). All in all, these models yielded excellent correlations between differentiation day and predicted “age.” After the second round to readjust coefficients, the Spearman’s rank correlations between predicted age and original age for biological replicates group A, B, C and D samples are 0.85, 0.86, 0.93 and 0.94, respectively.

## Discussion

AMD is a bilateral ocular condition especially noticed in older individuals resulting in irreversible vision impairment caused by the progressive loss of photoreceptors in the macula, a region at the center of the retina. In general, AMD risk is highly dependent upon factors such as a patient’s advanced age, familial history of AMD, signs and symptoms of pigmentary and drusen aberration, as well as lifestyle (e.g., drinking, smoking and diet) (62–64). Treatment for AMD is based upon disease category and stage. While there is no treatment in early stage AMD, regular check-ups, lifestyle changes, and close monitoring are important to initiate prompt treatment in case of occurrence of one of the late-stage forms (neovascular AMD). Monitoring is also important for intermediate AMD, as dietary supplements (minerals and vitamins) may decrease disease progression (26, 65). However, thus far, limited research had been performed using biological age clocks as endpoints for disease monitoring and therapeutic intervention. Therefore, to begin addressing this issue, we built regression age clocks for the retina and investigated their utilization in AMD cases with increasing severity. The clocks yielded good separation among AMD samples with increasing severity score (MGS1-4), regardless of whether clocks had been trained using retina tissues, dermal fibroblast samples, or combined datasets. Interestingly, cases with more advanced AMD pathology (MGS3 and MGS4) displayed more negative age acceleration. Furthermore, clock application to cultured fibroblasts, embryonic stem cells, and iPSC cells yielded the predicted behavior of the models. For instance, iPSCs displayed significant lower age than their fibroblast progenitors from 50-year-old donors. Finally, clock use in other relevant systems such as *in vitro* neuronal differentiation suggests new opportunities for broader applications.

Several genes have repeated occurrence across multiple clocks. Among the clock genes identified with high occurrence in our study, several (viz., *C16ORF72, GDF11, FBN2*) had been shown to play a role in aging, AMD, or other age-related diseases. For instance, Ratnapriya et al. (58) has previously identified *FBN2* (*Fibrillin 2*) gene’s impact in AMD (58). *FBN2* localizes to the Bruch’s membrane. The expression of *FBN2* gene decreases in aging as well as in AMD eyes. Different common/rare variants in *FBN2* can lead to early onset macular degeneration with Mendelian inheritance. In our analysis, we also identified the *C16ORF72* gene (60), which is also denoted Telomere Attrition and p53 Response 1 (*TAPR1*). It is well-known that telomere erosion in cells with insufficient amounts of telomerase reverse transcriptase (denoted as *TERT*) leads to age-associated tissue dysfunction as well as senescence at least partially through p53 (60). A genome-wide CRISPR screen in TAPR1-disrupted cells detected a relationship with TERT. Cells lacking TAPR1 or TERT displayed elevated p53 levels. Their transcriptional signatures are also consistent with the event of p53 upregulation. The higher p53 response in the TERT- or TAPR1-deficient cells are further exacerbated through treatment with the p53 stabilizer nutlin-3a as well as *MDM2* inhibitor, all of which coincide with a further deterioration of cell fitness. The regulation of p53 requires various context-specified controls that underlie its overall functionality (66). TAPR1 protects against deleterious telomere erosion or DNA damage through constraining p53. All in all, *C16ORF72/TAPR1* is a regulator of telomere integrity as well as p53 and appears to play a mechanistic role in aging.

Our analysis also identified Growth differentiation factor 11 (*GDF11*), a secreted factor in the TGFß family of cytokines (59, 61), as a key clock gene. It has long been suggested that GDF11 declines with increasing age and that the restoration of systemic GDF11 toward “young” levels could be advantageous for some age-related conditions. GDF11 is a complex rejuvenation factor in the aging cells. Some studies have reported the supplementation of GDF11 in skeletal muscle regeneration (67, 68). However, outcomes and possibly mode of action are poorly understood and seem dependent on GDF11 dose, with higher dose leading to the skeletal muscle atrophy as well as cachexia (69, 70). Other studies examined the effect of GDF11 gene and downstream ActRII pathway on cardiac activity and bone (59). GDF11 enhances the neurovascular disease as well as neurodegenerative disease, while it also enlarges the volume of the skeletal muscle and further improves the muscular strength (61). Its long-term biological impact might include the reversal of senescence in clinical aspects along with the capability for reversing age-related pathological variation and regulating the post-injury organ regeneration. Although some contradictions are still ongoing regarding the activities of GDF11, our identification of GDF11 as a key clock gene in the retina highlight its likely role in aging and age-related diseases.

Our study generates observations regarding retina age clock behavior and performance in AMD. One limitation of our study is that we only focused on training clocks with a single objective (reduction of MAE), while other factors (e.g., “gap between predicted and chorological age”) were not incorporated at this stage. In future, we envision extending this work through the inclusion of new feature selection strategies along with other machine learning approaches toward ever more useful biomarkers of aging and AMD. Furthermore, implementation and application of AMD targeted age clocks in screens for therapeutic intervention as well as clinical and population settings might be promising. The efforts could yield useful new therapeutics as well as improved tools for monitoring AMD risk and disease progression.

## Data Availability Statement

Retina (AMD) gene expression human data has been collected from NCBI Gene Omnibus ID: GSE115828, while dermal fibroblast gene expression data, primary fibroblast dataset and neuronal differentiation dataset can be retrieved with NCBI Gene Omnibus ID: GSE113957, GSE97265, and GSE56796, respectively.

## Ethics Statement

Ethical review and approval was not required for the study in accordance with the local legislation and institutional requirements.

## Author Contributions

SM and BL conceived and designed the study, analyzed the data, and wrote the manuscript. SM collected the data and wrote the programming code. FG, DB, and DV contributed to reagents and expertise. All authors edited the manuscript.

## Conflict of Interest

The authors declare that the research was conducted in the absence of any commercial or financial relationships that could be construed as a potential conflict of interest.

## Publisher’s Note

All claims expressed in this article are solely those of the authors and do not necessarily represent those of their affiliated organizations, or those of the publisher, the editors and the reviewers. Any product that may be evaluated in this article, or claim that may be made by its manufacturer, is not guaranteed or endorsed by the publisher.

## Supplementary Material

Supplementary Figure 1 Plots of clock evaluation metrics across the 105 models developed in retina samples (AMD MGS1, full feature set) and implemented on MGS1-4 samples. **(A)** Boxplot of MAE (sample wise), **(B)** line plot of Median Age Acceleration (MAA) per model and (C) line plot of MAE per model.

Supplementary Figure 2 Plots of clock evaluation metrics across 105 models developed in retina samples (AMD MGS1; conserved feature set) and implemented on MGS1-4 samples: **(A)** line plot of Median Age Acceleration (MAA) per model, **(B)** line plot of MAE per model, and **(C)** box plot of MAE using all retina (AMD MGS1 sample based) age models for the set of 5,321 common genes.

Supplementary Figure 3 Plots of clock evaluation metrics across 143 models developed in dermal fibroblast and implemented on MGS1-4 samples: **(A)** line plot of Median Age Acceleration (MAA) per model, **(B)** line plot of MAE per model, and **(C)** box plot of MAE using all dermal fibroblast-based age models for the conserved feature set (5,321 common genes).

Supplementary Figure 4 Plots of clock evaluation metrics across 248 models developed in dermal fibroblast and retina samples (AMD MGS1-based; conserved feature set) and implemented on MGS1-4 samples: **(A)** line plot of Median Age Acceleration (MAA) per model, **(B)** line plot of MAE per model, and **(C)** box plot of MAE using all joint models for the conserved feature set (5,321 common genes).

Supplementary Figure 5 Boxplots with age acceleration during neural differentiation **(A–D)**, sample group-wise boxplots of predicted time (in day) **(E–H)**, Regression plots **(I–L)** and sample group-wise line plots of MAE **(M–P)** for Group A, Group B, Group C and Group D samples, respectively. Retina age clocks were applied to neuronal differentiation without the readjustment of coefficient procedure on the retina (AMD MGS1) age models.

Supplementary Figure 6 Boxplots with age acceleration during neural differentiation **(A–D)**, sample group-wise boxplots of predicted time (in day) **(E–H)**, Regression plots **(I–L)** and sample group-wise line plots of MAE **(M–P)** for Group A, Group B, Group C and Group D samples, respectively, with readjustment of coefficient procedure on the retina (AMD MGS1) age models.
