# Supplementary material for: Novel Epigenetic Clock Biomarkers of Age-Related Macular Degeneration
Source: Front Med (Lausanne). 2022 Jun 16;9:856853. doi: 10.3389/fmed.2022.856853 (PMC9244395; doi:10.3389/fmed.2022.856853)
Supplement: Supplementary Figure 1 — Plots of clock evaluation metrics across the 105 models developed in retina samples (AMD MGS1, full feature set) and implemented on MGS1-4 samples. (A) Boxplot of MAE (sample wise), (B) line plot of Median Age Acceleration (MAA) per model and (C) line plot of MAE per model. [file Data_Sheet_1.PDF]

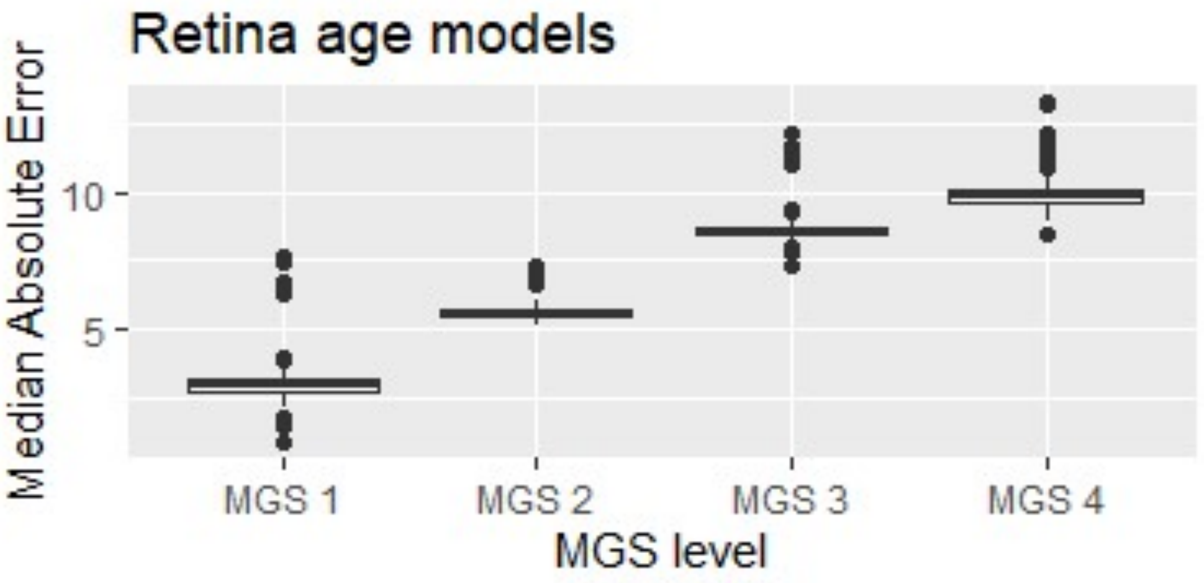

(A) Retina age models (full feature set)

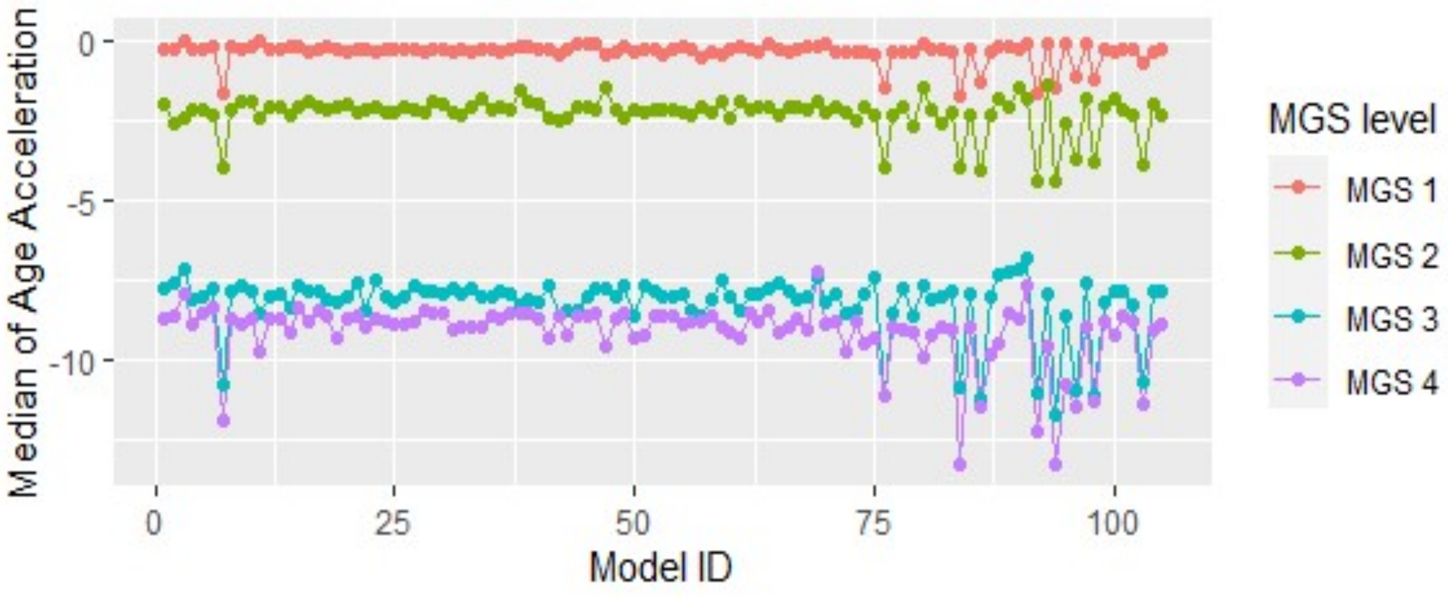

(B) Retina age models (full feature set)

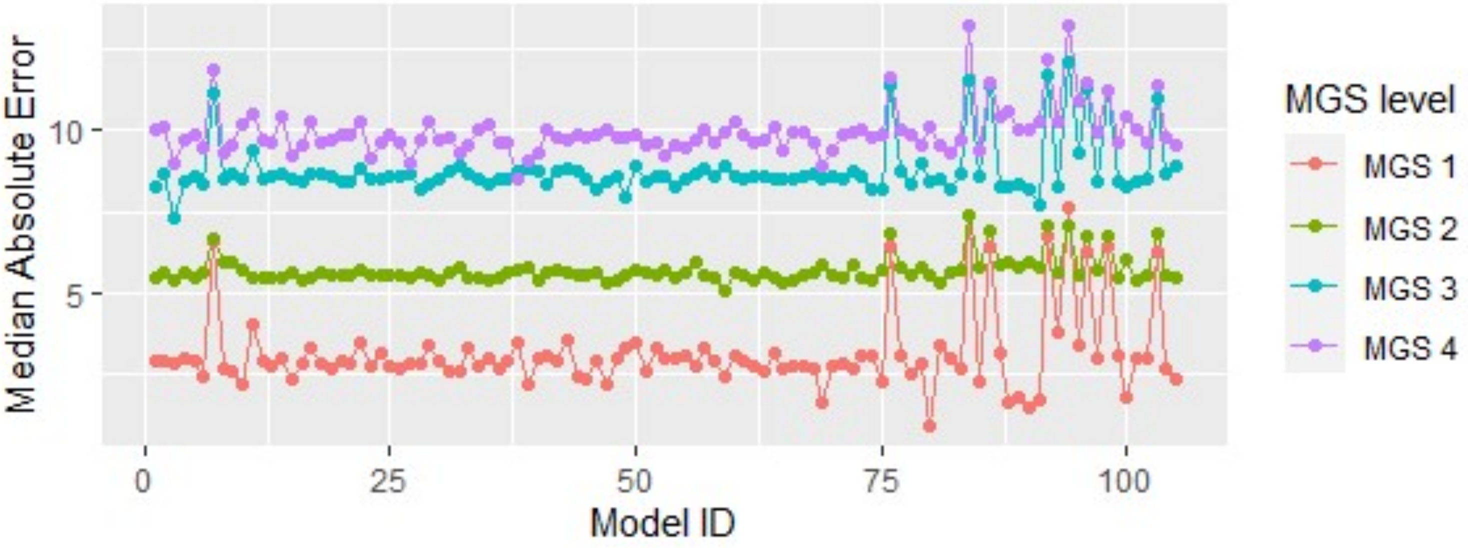

(C) Retina age models (full feature set)
