# Supplementary material for: Novel Epigenetic Clock Biomarkers of Age-Related Macular Degeneration
Source: Front Med (Lausanne). 2022 Jun 16;9:856853. doi: 10.3389/fmed.2022.856853 (PMC9244395; doi:10.3389/fmed.2022.856853)
Supplement: Supplementary Figure 2 — Plots of clock evaluation metrics across 105 models developed in retina samples (AMD MGS1; conserved feature set) and implemented on MGS1-4 samples: (A) line plot of Median Age Acceleration (MAA) per model, (B) line plot of MAE per model, and (C) box plot of MAE using all retina (AMD MGS1 sample based) age models for the set of 5,321 common genes. [file Data_Sheet_2.PDF]

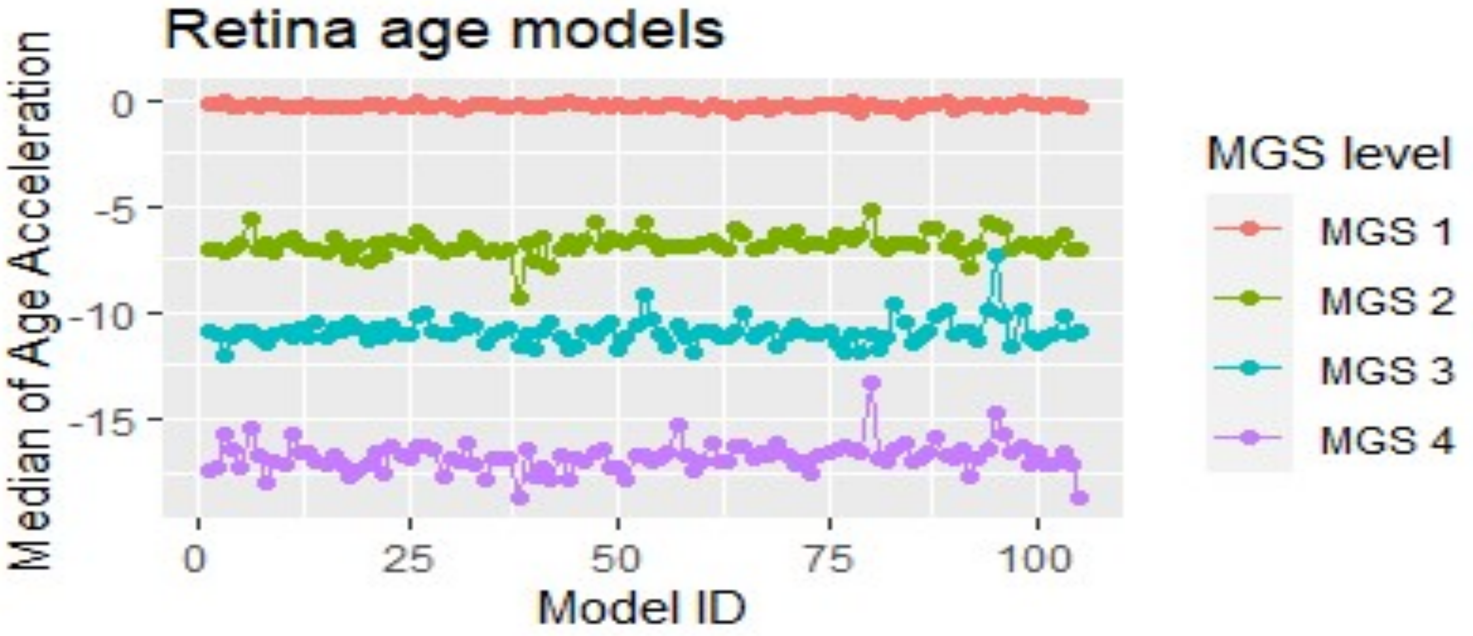

(A) Retina age models (conserved feature set)

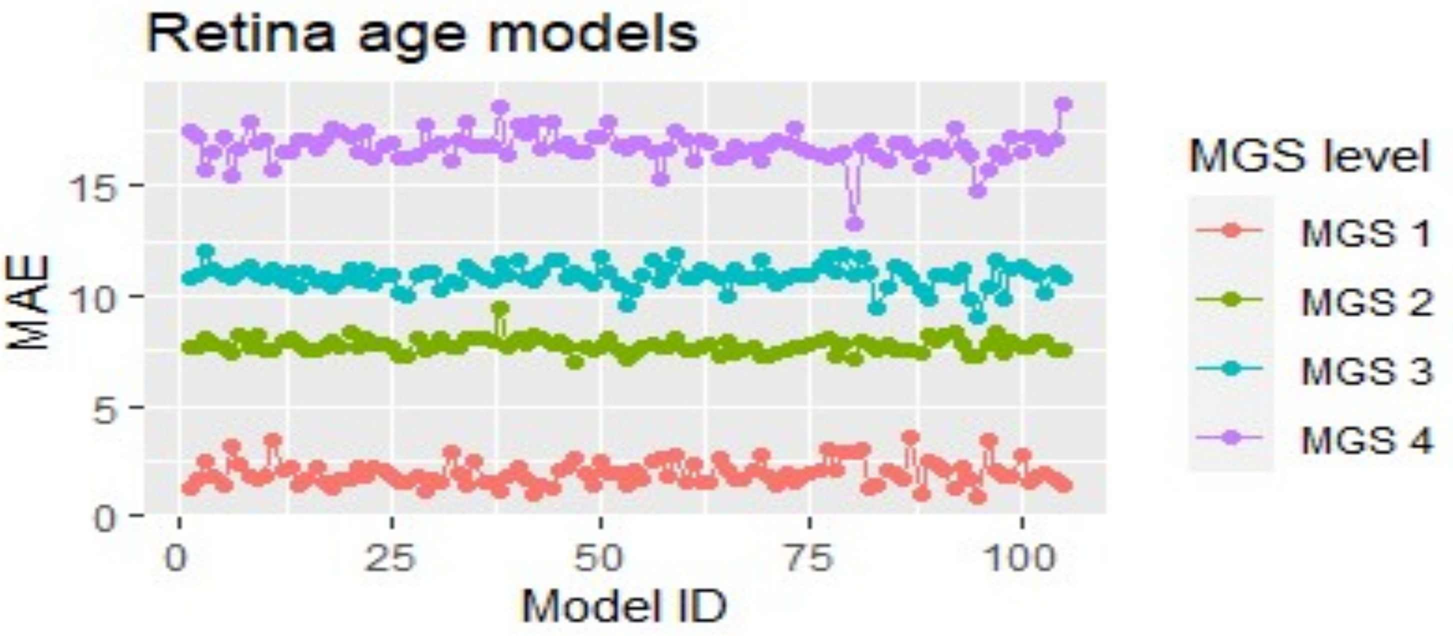

(B) Retina age models (conserved feature set)

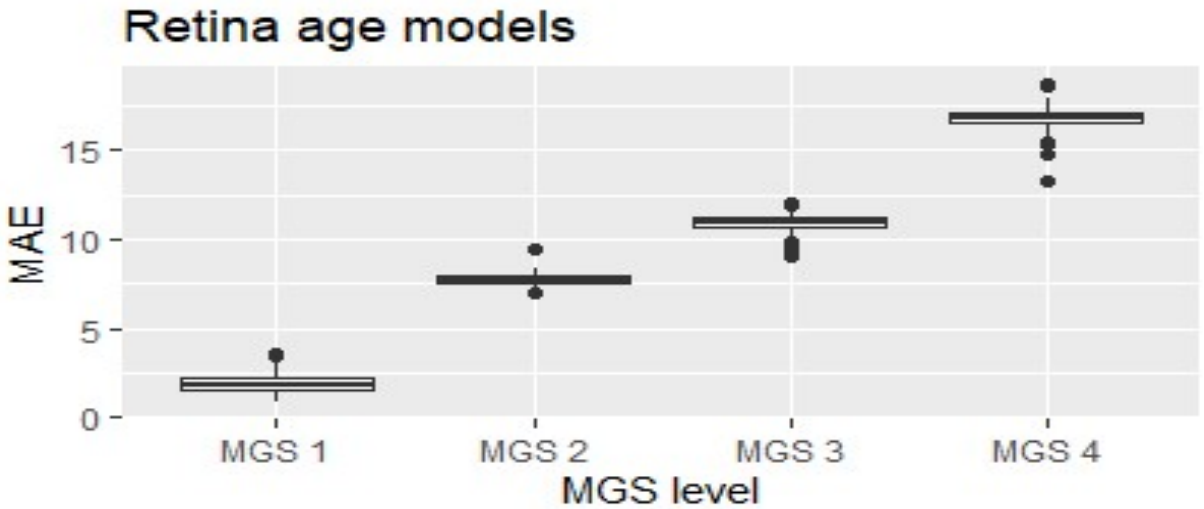

(C) Retina age models (conserved feature set)
