# Supplementary material for: Novel Epigenetic Clock Biomarkers of Age-Related Macular Degeneration
Source: Front Med (Lausanne). 2022 Jun 16;9:856853. doi: 10.3389/fmed.2022.856853 (PMC9244395; doi:10.3389/fmed.2022.856853)
Supplement: Supplementary Figure 3 — Plots of clock evaluation metrics across 143 models developed in dermal fibroblast and implemented on MGS1-4 samples: (A) line plot of Median Age Acceleration (MAA) per model, (B) line plot of MAE per model, and (C) box plot of MAE using all dermal fibroblast-based age models for the conserved feature set (5,321 common genes). [file Data_Sheet_3.PDF]

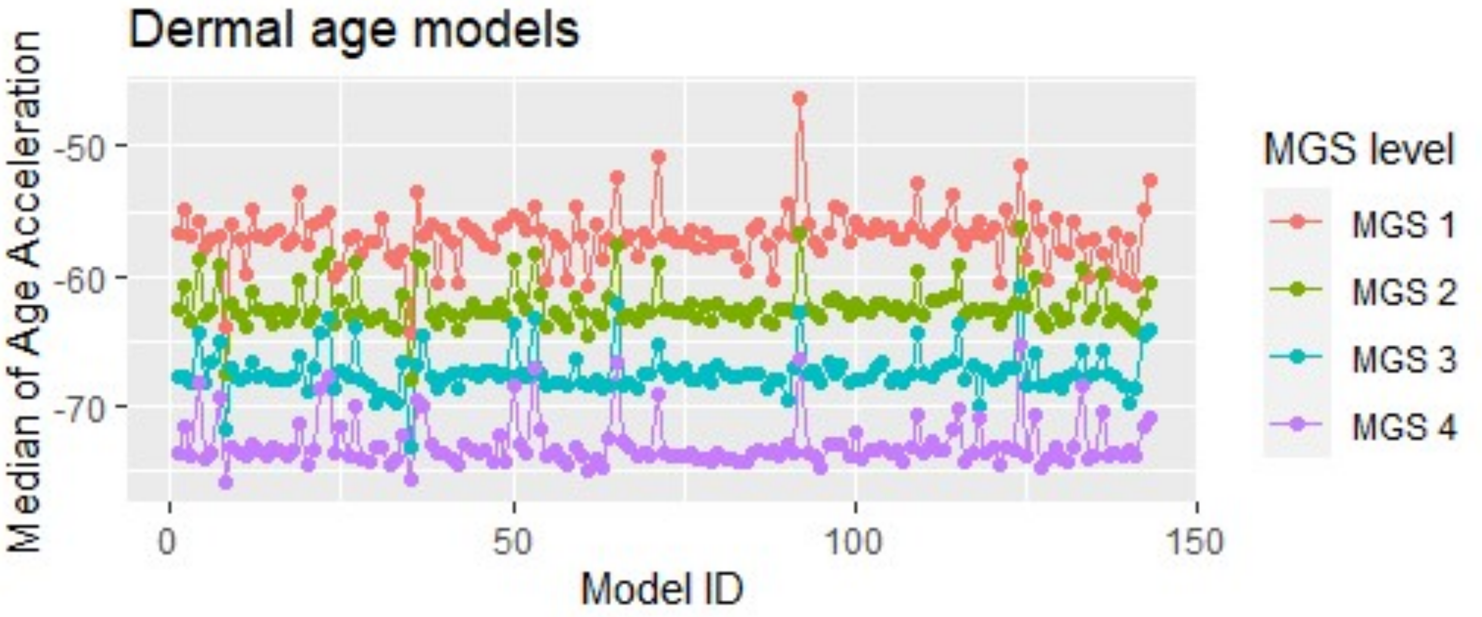

(A) Dermal age models (conserved feature set)

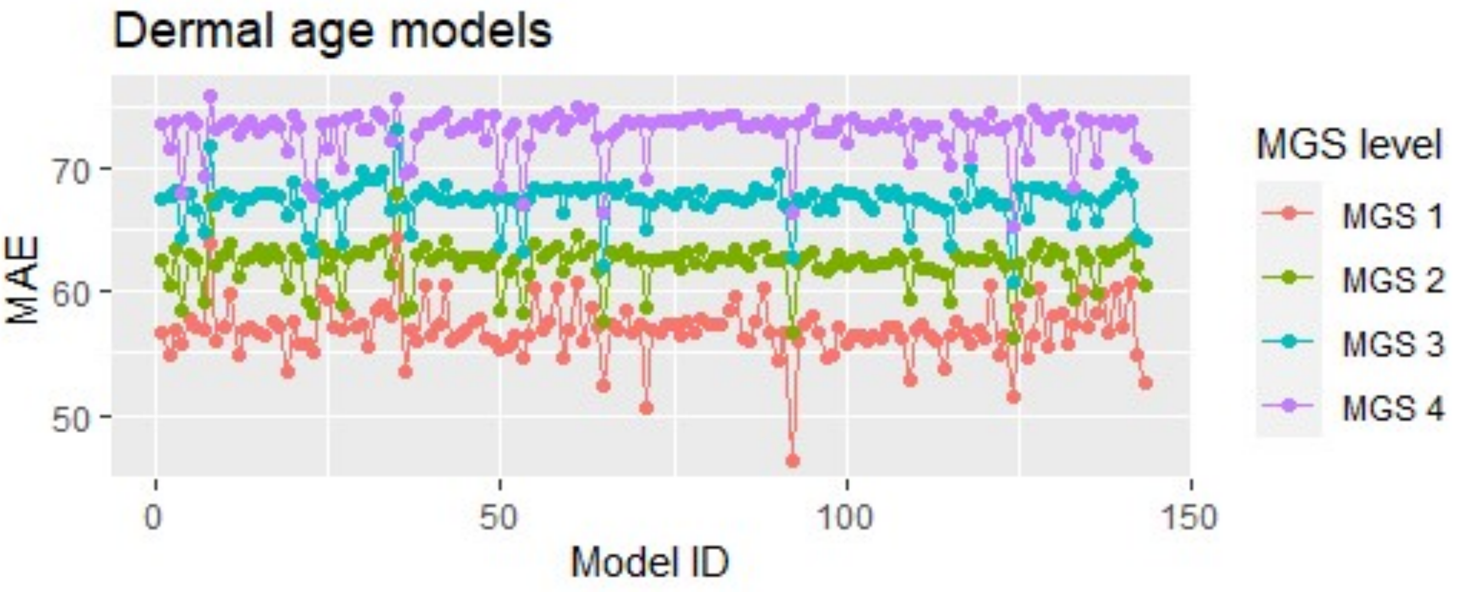

(B) Dermal age models (conserved feature set)

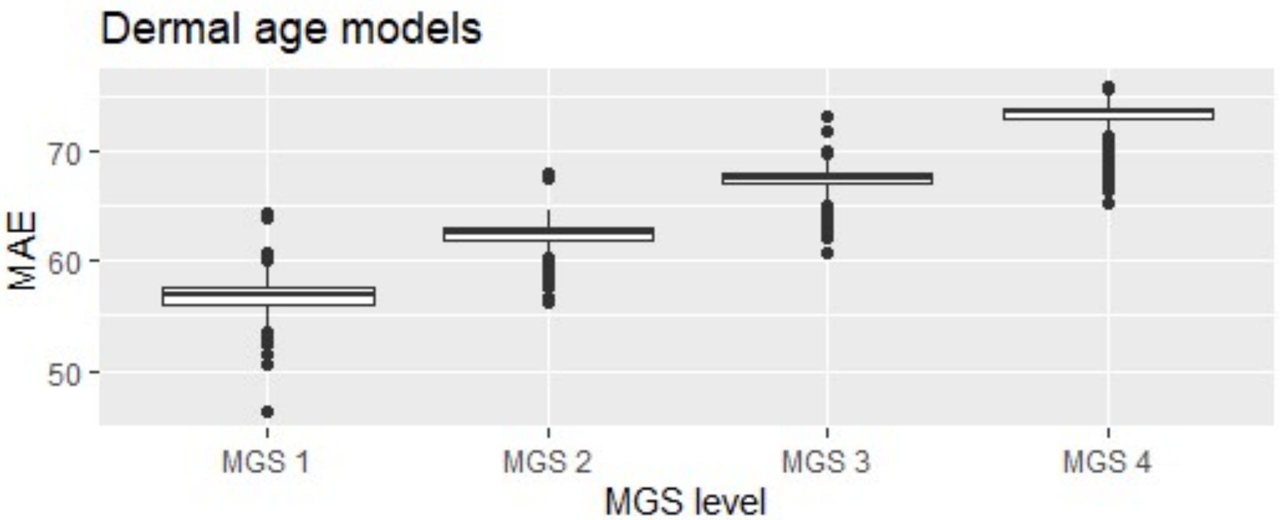

(C) Dermal age models (conserved feature set)
