# Supplementary material for: Novel Epigenetic Clock Biomarkers of Age-Related Macular Degeneration
Source: Front Med (Lausanne). 2022 Jun 16;9:856853. doi: 10.3389/fmed.2022.856853 (PMC9244395; doi:10.3389/fmed.2022.856853)
Supplement: Supplementary Figure 4 — Plots of clock evaluation metrics across 248 models developed in dermal fibroblast and retina samples (AMD MGS1-based; conserved feature set) and implemented on MGS1-4 samples: (A) line plot of Median Age Acceleration (MAA) per model, (B) line plot of MAE per model, and (C) box plot of MAE using all joint models for the conserved feature set (5,321 common genes). [file Data_Sheet_4.PDF]

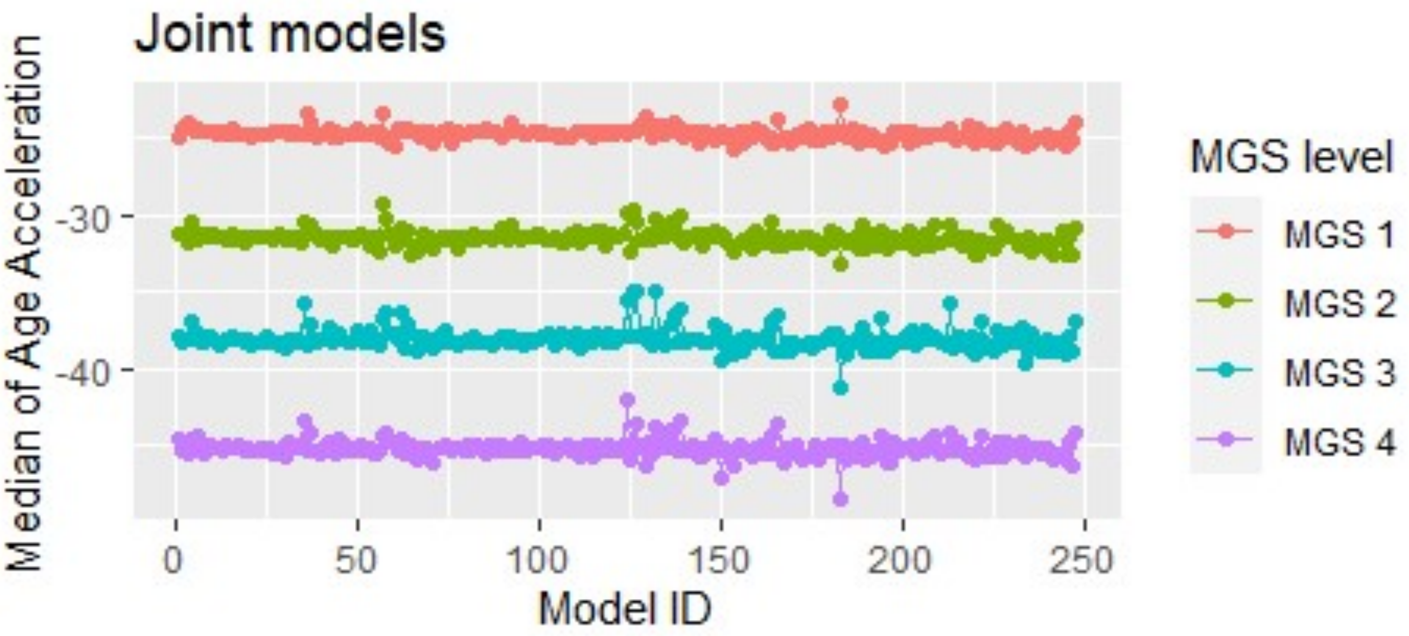

(A) Joint models (conserved feature set)

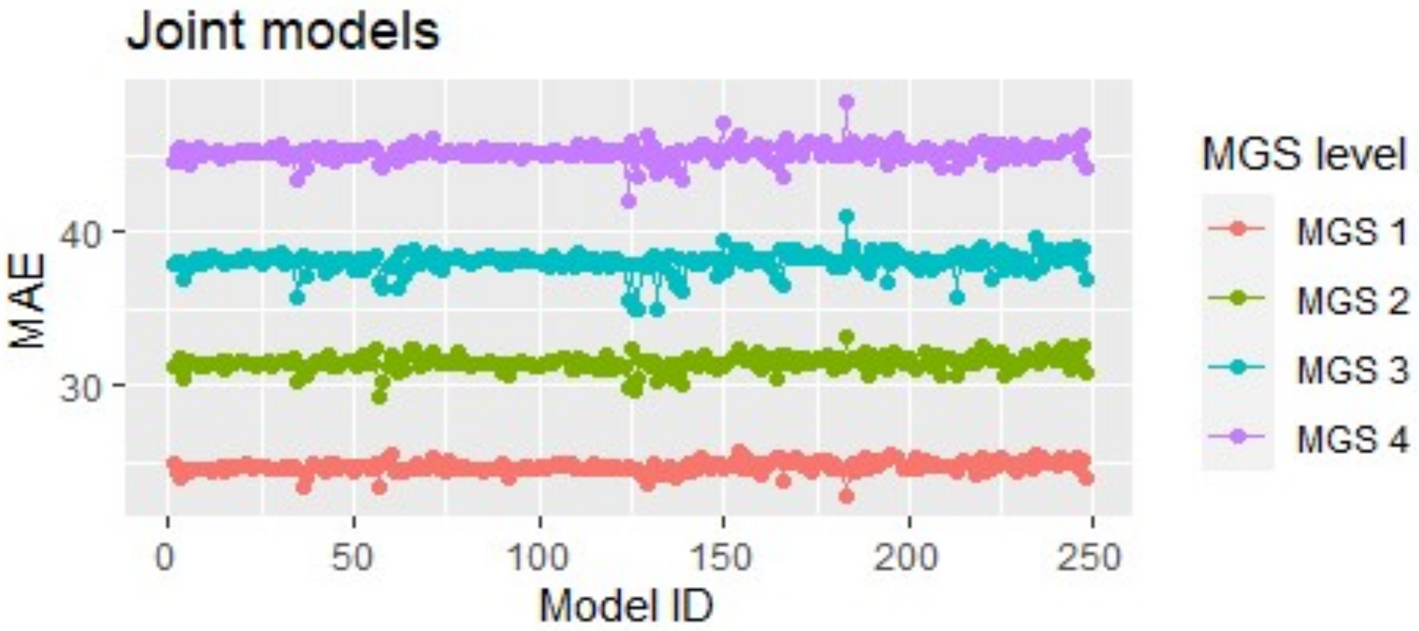

(B) Joint models (conserved feature set)

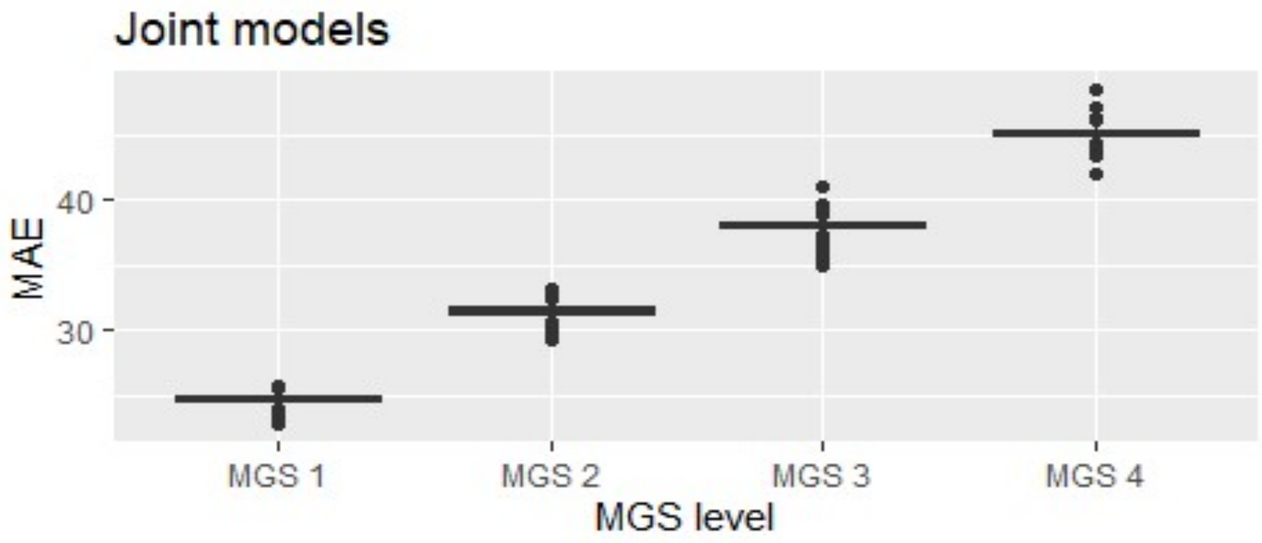

(C) Joint models (conserved feature set)
