# Supplementary material for: Novel Epigenetic Clock Biomarkers of Age-Related Macular Degeneration
Source: Front Med (Lausanne). 2022 Jun 16;9:856853. doi: 10.3389/fmed.2022.856853 (PMC9244395; doi:10.3389/fmed.2022.856853)
Supplement: Supplementary Figure 5 — Boxplots with age acceleration during neural differentiation (A–D), sample group-wise boxplots of predicted time (in day) (E–H), Regression plots (I–L) and sample group-wise line plots of MAE (M–P) for Group A, Group B, Group C and Group D samples, respectively. Retina age clocks were applied to neuronal differentiation without the readjustment of coefficient procedure on the retina (AMD MGS1) age models. [file Data_Sheet_5.PDF]

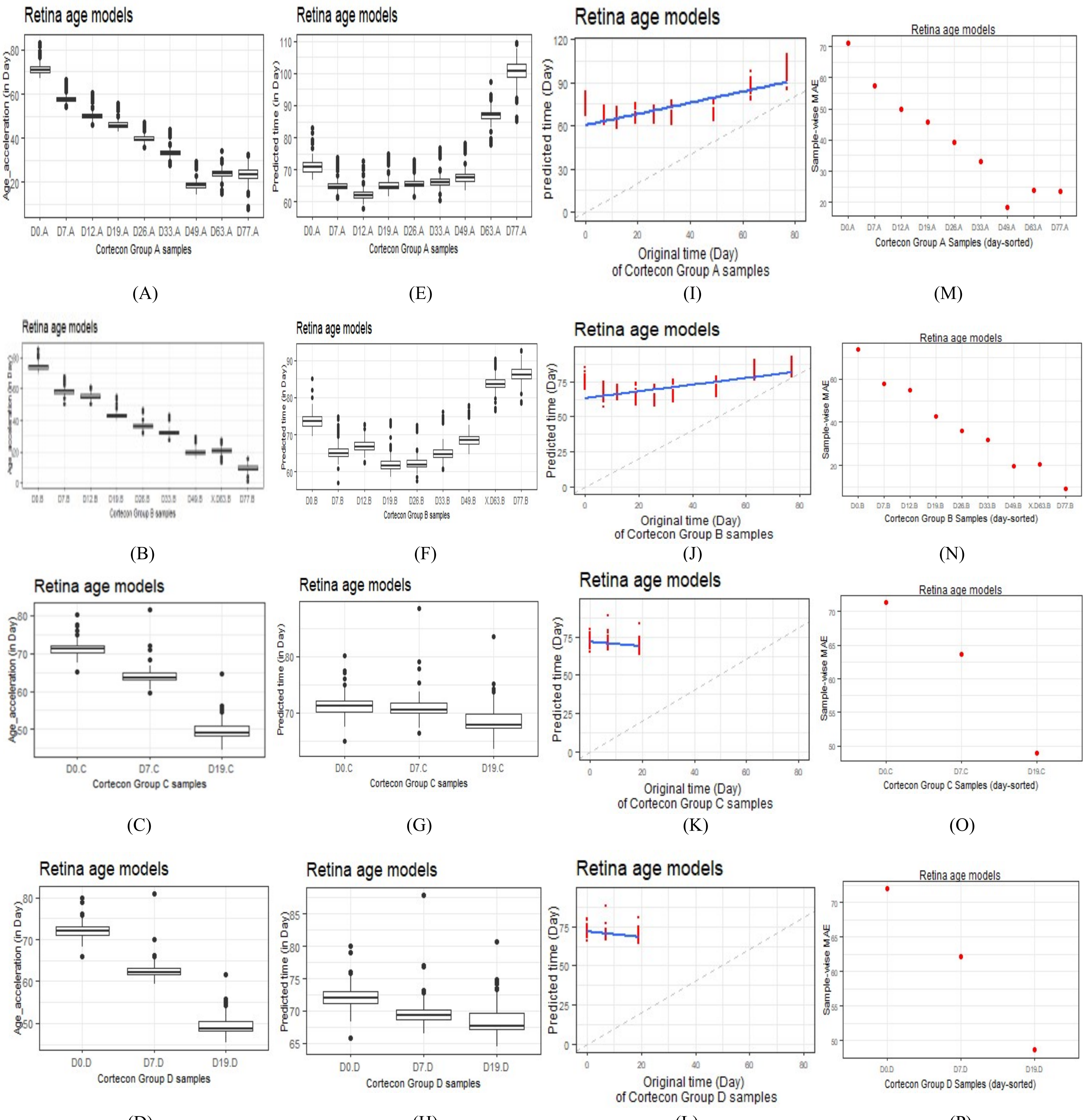

(A) (E) (I) (M)

(B) (F) (J) (N)

(C) (G) (K) (O)

(D) (H) (L) (P)
