# Supplementary figures and images for: Novel Epigenetic Clock Biomarkers of Age-Related Macular Degeneration
Source: Front Med (Lausanne). 2022 Jun 16;9:856853. doi: 10.3389/fmed.2022.856853 (PMC9244395; doi:10.3389/fmed.2022.856853)

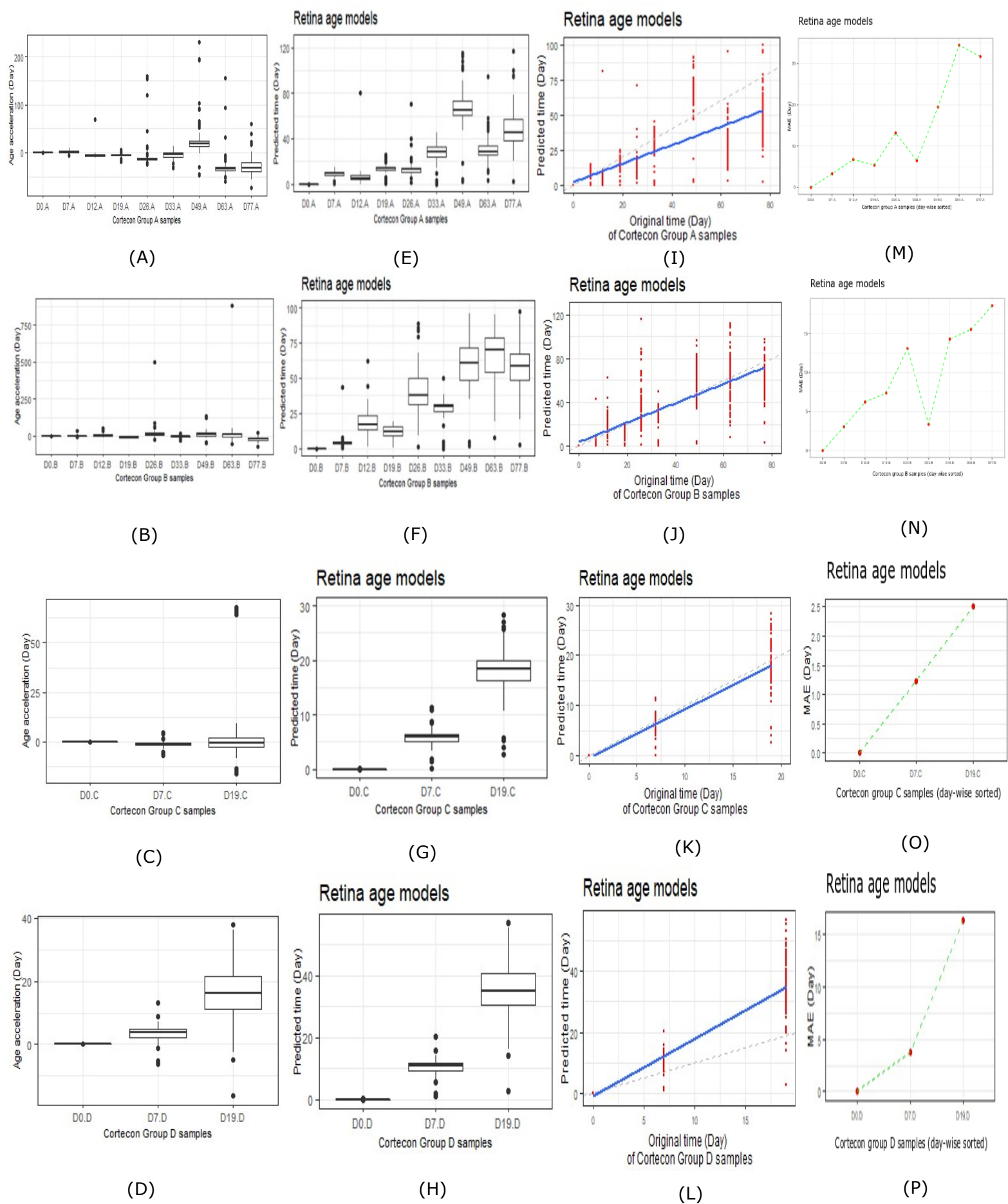

Supplement: Supplementary Figure 6 — Boxplots with age acceleration during neural differentiation (A–D), sample group-wise boxplots of predicted time (in day) (E–H), Regression plots (I–L) and sample group-wise line plots of MAE (M–P) for Group A, Group B, Group C and Group D samples, respectively, with readjustment of coefficient procedure on the retina (AMD MGS1) age models. [file Data_Sheet_6.PDF]
